# Ethics, Economics, and the Use of Primaquine to Reduce Falciparum Malaria Transmission in Asymptomatic Populations

**DOI:** 10.1371/journal.pmed.1001704

**Published:** 2014-08-19

**Authors:** Yoel Lubell, Lisa White, Sheila Varadan, Tom Drake, Shunmay Yeung, Phaik Yeong Cheah, Richard J. Maude, Arjen Dondorp, Nicholas P. J. Day, Nicholas J. White, Michael Parker

**Affiliations:** 1 Mahidol-Oxford Tropical Medicine Research Unit, Faculty of Tropical Medicine, Mahidol University, Bangkok, Thailand; 2 Centre for Tropical Medicine, Nuffield Department of Medicine, University of Oxford, United Kingdom; 3 International Commission of Jurists, Geneva, Switzerland; 4 Faculty of Public Health and Policy, The London School of Hygiene & Tropical Medicine, London, United Kingdom; 5 The Ethox Centre, Nuffield Department of Population Health, University of Oxford, United Kingdom

## Abstract

Yoel Lubell and colleagues consider ethical and economic perspectives on mass drug administration of primaquine to limit transmission of *P. falciparum* malaria.

*Please see later in the article for the Editors' Summary*

**Summary Points**

- Rapidly achieving falciparum malaria elimination could require mass antimalarial treatment of asymptomatic individuals to eliminate the parasite reservoir that sustains malaria transmission.
- Primaquine is the only licenced antimalarial that kills mature *Plasmodium falciparum* gametocytes, but it is associated with a dose-dependent risk of haemolysis in G6PD-deficient individuals.
- We discuss ethical and economic considerations pertaining to mass primaquine administration in malaria elimination programmes, which go beyond those encountered in other public health interventions. These include the lower direct benefit for individuals at higher risk, the increasingly available diagnostic tests for G6PD deficiency, and the economic implications of testing.
- We propose a research agenda to assist informed and rational policy decision making in the rollout of primaquine mass drug administration that is pragmatically and economically viable and within acceptable ethical standards.

## Costs, Benefits, and Risks on the Pathway to Malaria Elimination

Following a decade of gains in malaria control, two divergent prospects are emerging: malaria elimination in many endemic countries, and spreading artemisinin-resistant *P. falciparum* undermining these gains. The potential benefits of malaria elimination are substantial, including the direct burden averted and economic growth through improved educational attainment and productivity; these gains were estimated recently to far outstrip the costs required to achieve them [1].

Much of the international investment in malaria control, approximating US$2.5 billion annually, is allocated to distributing insecticide treated nets (ITN) and improved diagnosis and treatment of clinical cases. While these contributed to the declining burden of malaria, avoiding the spread of artemisinin resistance and a final push to elimination could require additional interventions, notably population-wide antimalarial treatment.

There is a qualitative difference between interventions such as ITN and improved case management, offering immediate health gains to recipients, and the treatment of asymptomatic, often uninfected individuals for a broader community gain, which offers little or no direct benefit and may even harm recipients. An unfavourable individual risk/benefit ratio could deter both policy makers from adopting what could be a critical component of an effective elimination campaign, and health providers from supporting its effective implementation.

Mass drug administration (MDA) could, however, be essential to malaria elimination. This is because of the substantial proportion of malaria infections that are asymptomatic and chronic at densities lower than detection thresholds for microscopy or malaria rapid tests, even in areas of low malaria transmission. These infections still produce gametocytes, and in low-endemic settings are estimated to account for 20%–50% of transmission episodes [2]–[6]. Targeting asexual parasites in these carriers using drugs such as artemisinin combination therapies will limit de-novo gametocyte development but will not kill gametocytes already present. An MDA which includes a gametocytocidal drug might therefore be more effective than one which does not. High coverage would be key to its success.

Primaquine is the only licenced antimalarial that kills mature *P. falciparum* gametocytes, but safety concerns in individuals with glucose-6-phosphate dehydrogenase deficiency (G6PDd) have deterred its use to interrupt transmission on a large scale [3],[5],[7]. The X-linked G6PDd has gene frequencies ranging from 3% to 30% in malaria-endemic areas and causes dose-dependent haemolysis following administration of primaquine or other oxidant drugs [8].

Over two hundred million people have received primaquine, but in the majority of cases primaquine was given in a two-week course (0.25 mg base/kg/day) to prevent relapses of vivax malaria. This carries a substantially greater risk than single-dose administration to prevent transmission of falciparum malaria. Excluding Chinese data for which denominators are unclear, it has been estimated that the risk of death associated with use of primaquine is one in 621,428 individuals treated, with an upper 95% CI boundary of one in 407,807 [9]. A review of primaquine MDA aiming for vivax elimination found very few reported incidences of severe adverse events following multiple primaquine doses [10]. The highest rate was in Azerbaijan where, despite a high prevalence of severe G6PDd variants, only seven out of 30,000 individuals treated had a severe adverse event, none of whom required hospitalization. Only one death has been reported following a single dose of primaquine to block *P. falciparum* transmission, and there have been occasional reports of significant haemolysis following the 0.75 mg base/kg gametocytocidal dose hitherto recommended. The WHO recently adjusted its guidelines for the management of falciparum malaria in low-transmission settings to allow for a single, low dose of primaquine (0.25 mg base/kg) without G6PDd testing, except for pregnant women and infants <1 year of age [11]. This was considered highly unlikely to induce clinically significant haemolysis even in severely G6PD-deficient individuals, concluding that the transmission-blocking benefit outweighed the associated risk. The effectiveness of adding a single low-dose primaquine to MDA depends on several factors, and, in settings where the other drugs are highly effective and coverage is very high, it would add little. This Essay assumes that this addition would be effective and examines the ethical and economic arguments for and against the use of primaquine and G6PD testing in MDA.

## Unique Challenges in Use of Primaquine as a *P. falciparum* Gametocytocide in MDA

Previous public health interventions, such as the smallpox and polio eradication campaigns, posed comparable risks to vaccinated individuals, as does the use of sulfadoxine–pyrimethamine and other antimalarials in intermittent preventive treatment often provided to children and pregnant women in malaria-endemic regions [12]–[14]. The use of primaquine in MDA introduces additional unique challenges in several important respects. In terms of the risks involved, compared with smallpox and eradication campaigns, a failed MDA programme could cause significant harm if it contributed to emerging drug resistance and a resurgence of malaria in populations with lowered immunity.

Ethically, there are two additional aspects that make use of primaquine as a *P. falciparum* gametocytocide in MDA contentious. Firstly, the use of primaquine offers no curative or prophylactic effect to the individuals receiving it (other than the indirect benefit of reducing the likelihood of future malaria), but it does expose them to risk of harm (albeit substantially less than with higher doses, or with longer courses as used to eliminate *P. vivax*). Asymptomatic carriers targeted by the programme are less likely to benefit from the programme because of their immunity to malaria.

Secondly, the technology to identify G6PD-deficient individuals and exclude them from treatment is increasingly available. While intuitively appealing, this involves significant trade-offs – it would lower gametocytocidal treatment coverage, jeopardising the campaign and therefore increasing the probability of a rebound in malaria cases; and it requires significant resources to prevent what is likely be a very low degree of harm. Assuming a cost of US$2 per test, the above estimated mortality of 1–2 million, and a life expectancy of 20 years, would result in a cost per DALY averted in the range of US$50,000 to US$100,000, far exceeding what is normally considered cost-effective in low-income settings. The substantially lower estimated risk associated with single-dose primaquine would imply an even higher cost per DALY averted. This investment, from a consequentialist perspective, might be unethical, if these resources could be used elsewhere to generate far greater health benefits; but from other ethical perspectives and given the unique context, use of the tests could still be considered an ethical obligation.

Taken together, the decision to implement MDA with primaquine introduces challenges both within public health ethics and between public health ethics and those of clinical practice [15],[16].

## The Public Health Perspective

At the core of public health ethics is the ambition of improving the health of populations whilst minimising potential risks to the liberty or autonomy of individuals [14]. A simple “back of the envelope” consequentialist assessment suggests that, on average, the risk of malaria mortality in low-transmission settings is at least several hundred-fold that of a fatal adverse event following low-dose primaquine (based on the 2012 World Malaria Report annual malaria mortality rate of 0.02% in endemic areas and an estimated risk of primaquine-induced mortality equal to or lower than 1–2 per million, derived largely from the higher dosages used for the radical cure of vivax malaria [9],[10]). It is important to note that for vivax malaria the risk/benefit ratio of primaquine MDA will be very different, as although there may be individual benefit from prevention of relapse, the required primaquine dose and the associated risk is much higher, while the risks associated with vivax malaria are lower, in which case the consequentialist assessment will be far less favourable.

Despite a favourable consequentialist assessment, an unequal distribution of risk amongst different ethnic groups and marginalized communities with poorer access to medical care to manage adverse events poses further challenges. The confluence of malaria transmission, marginalized communities, and in some instances higher prevalence of G6PDd is a reality in many endemic areas [17]. Central government and international public health officials will have to ensure a balanced approach that does not infringe on individual and community rights when pursuing high coverage, as was reportedly the case in the final stages of smallpox eradication [18]. Maintaining trust between the public and health-care workers should remain a priority. The potential for perceived serious adverse events in healthy individuals, whether or not they are truly related to the drug, could jeopardise this relationship.

From the public health planners' perspective, the question of consent on the part of recipient communities is paramount. There is a distinction between medical research, which usually necessitates an opt-in informed consent process, and public health interventions where an opt-out approach might be more appropriate [19]. In extreme (and contentious) instances it has even been considered acceptable to take compulsive measures to avoid the spread of infectious diseases, such as interning tuberculosis patients to complete their treatment [20]. The spread of artemisinin-resistant *P. falciparum* to India and Africa could undermine the recent global gains in malaria morbidity and mortality [21]. Stopping it should have the highest priority. There are compelling reasons, however, for strong community engagement programmes that seek to achieve community awareness and approval for MDA. From an ethical standpoint, given the absence of individual immediate benefit from the primaquine component and the potential harm, informed community and individual consent is particularly relevant. Furthermore, from a pragmatic perspective it will be impossible to achieve the required high coverage without strong local support.

In considering these challenges, reference should be made to relevant national and international legal frameworks and ethics guidelines. Such laws, embedded in national constitutions and legislative mechanisms, as well as conventions and customary obligations under international law, guarantee specific legal entitlements to individuals subjected to medical interventions, even where these are at the expense of the “greater good” [22].

## The Health-Care Provider Perspective

Although health providers administering treatments in an MDA programme might be committed to reducing malaria transmission in the population, their primary concern is likely to be the well-being of individuals in their care. Indeed, health-care professionals have a legal and moral duty to act in the best interests of the patient. In practical terms, this means that health-care professionals exercising their duty of care will generally administer medication only where the risk of harm is minimal or where the individual benefit outweighs the risk of harm [23].

This is *prima facie* at odds with the administration of a potentially harmful drug to an asymptomatic or even uninfected individual for the benefit of the wider community. Much depends, however, on the degree of risk involved. For therapeutic interventions the obligation to ensure informed consent is proportional to the risk involved [24],[25]; in a public health context it could be contended that disclosure requirements should be higher, especially where there is no direct health benefit. Assuming a community engagement programme has adequately informed recipients of the risks and benefits involved, a key question from the provider perspective remains the extent to which consent from the recipient (implicit or explicit) provides sufficient normative justification to administer the intervention. From their perspective consent on behalf of the recipient is necessary but not in itself sufficient to act in accordance with clinical ethics [25], unless they are equally convinced the benefits outweigh the risks. Engagement of health-care providers in the planning and execution of the programmes to ensure their support is therefore imperative.

Even if implicit informed consent is normatively acceptable, ensuring its validity will be challenging. Will individuals comprehend the difference between a public health intervention and an individual curative treatment [26]? Will they find it difficult to object to such treatment, either because they are grateful to, or overly respectful towards, health-care providers? Will peer pressure place undue influence on them, particularly if it is understood that refusal to participate could jeopardise the initiative for which others have accepted the risk? These questions are of particular concern amongst marginalized communities.

## Possible Ways Forward

Considering these challenges, how best to proceed? One option is to continue minimising the burden of clinical cases without pushing forward with an aggressive elimination campaign. This will spare the resource-intensive phase required in stamping out final infections and avoiding re-importation, as well as potential harm associated with measures such as primaquine MDA. The risk here is that the spread of artemisinin resistance undermines our ability to effectively treat clinical cases coupled with a rebound in transmission, resulting in a large increase in morbidity and mortality. The global impact could be enormous.

With a more ambitious objective of interrupting transmission, an alternative strategy targeting asymptomatic infections and circumventing the ethical challenges in MDA is to screen for parasitaemia and treat only positive individuals, offering a clearer benefit to drug recipients. The malaria tests available for wide-scale screening, however, often fail to identify low levels of parasitaemia [27]. As such, a large proportion of asymptomatic carriers – the primary target of this intervention – would be missed, potentially rendering the programme ineffective.

Given these limitations, MDA with single dose primaquine might be the only option for successful elimination campaigns. The risk associated with this low dose is small, even in G6PD-deficient individuals, and while its existence raises ethical questions both in relation to public health and to clinical care, these can be managed with careful consideration. On this basis it is our view that MDA programmes that include the use of primaquine are justifiable, but will require carrying out a significant programme of research together with implementation (see Box 1). A research and implementation agenda for primaquine MDA will require consideration of overall societal benefit against the obligation to protect individuals and minority groups from harm, as well as developing models of good practice for ensuring effective mechanisms for obtaining valid consent and respecting the duty of care owed to a patient by the health-care professional. These measures should be considered not only an ethical obligation, but a practical necessity to ensure high levels of compliance, key to the programmes' success.

**Box 1. A Research Agenda Prior to Policy Decisions on Rolling Out Primaquine MDA**

1. A number of MDA studies with single-dose primaquine and other drug regimens have been carried out or are underway; further studies are needed to inform on the impact on transmission and establish the regimens' safety profiles in different age groups and in G6PD normal and deficient subgroups.
2. These findings can be used in economic–epidemiological models to estimate the costs and benefits of MDA with or without gametocytocidal drugs and the probability and timeframe to elimination. Such models can also estimate the minimal coverage required for successful elimination, and explore the impact of excluding G6PD-deficient individuals from gametocytocidal treatment.
3. If exclusion of G6PD-deficient individuals is predicted to critically compromise the programme's effectiveness or the cost is prohibitive, further assessment should be undertaken to explore whether provision of primaquine without G6PD testing would comport with national and international legal obligations and comply with ethical standards.
4. Qualitative research is required to provide insight into the practical ethical challenges that may arise for health professionals and for community members. For example, it is important to understand community views about taking drugs when ill and when not; attitudes towards Western medicine; and previous experience with MDA programmes and any challenges associated with them.
5. Drawing on these findings, programme designs should be trialled to identify those that achieve the highest levels of coverage while complying with ethical and legal standards.

## Abbreviations

G6PD: glucose-6-phosphate dehydrogenase
ITN: insecticide-treated net(s)
MDA: mass drug administration
